# Editorial: Advances in grapevine genetic improvement: Towards high quality, sustainable grape production

**DOI:** 10.3389/fpls.2022.1080733

**Published:** 2022-11-14

**Authors:** Gabriella De Lorenzis, Pablo Carbonell-Bejerano, Silvia Laura Toffolatti, Javier Tello

**Affiliations:** ^1^ Dipartimento di Scienze Agrarie ed Ambientali, Università degli Studi di Milano, Milan, Italy; ^2^ Departamento de Viticultura, Instituto de Ciencias de la Vid y del Vino Consejo Superior de Investigaciones Científicas (CSIC), Universidad de La Rioja (UR), Gobierno de La Rioja, Logroño, Spain

**Keywords:** fruit quality, abiotic stress, biotic stress, breeding, phenotyping, target genes, CRISPR/Cas9

Grapevine (*Vitis vinifera* ssp. *vinifera*) is the fruit crop with the largest economic value worldwide, considering its derived products. However, emerging climate change-derived threats along with established pathogens compromise the sustainability of traditional viticultural systems. Grapevine genetic improvement is critical to face this situation, as well as to adapt to novel market needs and regulatory frameworks. Current breeding and selection programs focus on the obtention and exploitation of cultivars combining high quality fruit traits, adequate yield, and some level of resistance to major biotic and abiotic stressors. To this aim, two main activities are conducted: (i) the screening of today’s standing *Vitis* diversity (Wolkovich et al., 2018), and (ii) the generation of individuals gathering favorable traits (Töpfer and Trapp, 2022). Among the tools facilitating the success of these activities, the identification of genotypes harboring favorable alleles, and the knowledge on the underlying genomic regions and gene variants is basic to speed up current and future grapevine improvement activities (**Figure 1**).

**Figure 1:**
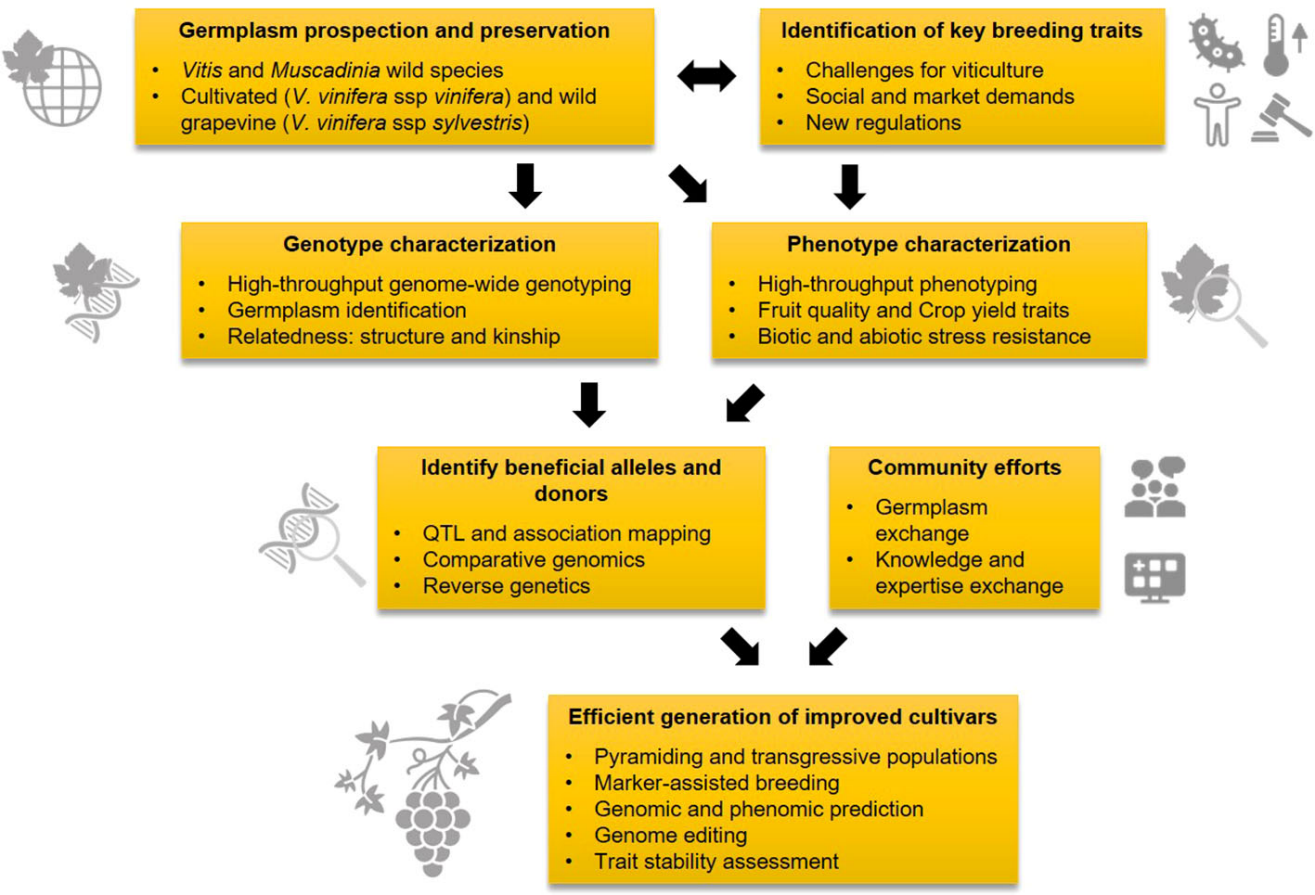
Workflow chart of grapevine genetic improvement activities towards high quality and sustainable grape production.

This Research Topic was aimed at collecting current findings on genetic strategies fostering grape production improvement for different purposes (e.g.: wine, table grapes, raisins, juice), as well as at shedding light on the genetic mechanisms involved in grape quality and adaptation to biotic and abiotic stress traits. It comprises five original research articles, one review article and one perspective article, which can be grouped into the following three major topics:

## Improving grape resistance to pests and diseases

Grapevine improvement to increase grape resistance to pests and diseases can rely on both conventional and modern breeding activities. Conventional breeding (classical or traditional breeding) starts from the selection of beneficial individuals from a crossing population, commonly generated through the cross between a susceptible *V. vinifera* parental genotype and a resistant (or tolerant) non-*vinifera* parental genotype (Vezzulli et al., 2022). This approach can take advantage of the modern tools capable of analyzing thousands of genetic markers in a high-throughput manner. Then, genetic variation can be associated with phenotypic variation (by QTL (Quantitative Trait Loci) or GWAS (Genome-Wide Association Study) approaches) to uncover the genetic architecture of the trait/s of interest. The identification of the genetic markers linked to the phenotype of interest allows the screening of large plant populations and progenies to select the individuals harboring beneficial alleles, *via* Marker-Assisted Selection (MAS). Combining disease severity ratings and information on 2,000 genetic markers, Karn et al. (2021) identified a new QTL derived from *Vitis aestivalis* responsible for the resistance to *Erysiphe necator*, causal agent of powdery mildew. The new locus is named *REN11* and it is located on chromosome 15. The authors suggested that the markers flanking *REN11* can be directly used for MAS. On the other hand, modern breeding is the process through which new varieties are developed applying genetic modification and genome editing techniques. Genome editing *via* CRISPR/Cas9 system is a powerful technique making possible different types of genetic modifications, such as insertion, deletion, or mutation. Olivares et al. (2021) proved the use of the CRISPR/Cas9 technology to knock-out four putative grapevine susceptibly genes to fungal diseases (*E. necator* and *Botrytis cinerea*) in cv. ‘Thompson Seedless’: *VviAIR12*, *VviSWEET4*, *VviLIN2*, and *VviDEL1*. Authors found that a *VviDEL1*-edited line showed a reduced susceptibility to *E. necator* infection, supporting the role of *VviDEL1* on grapevine resistance mechanisms against this fungal agent.

Phenotyping is fundamental for the discovery and exploitation of QTLs that are associated with disease resistance. However, as highlighted by Possamai and Wiedemann-Merdinoglu (2022), phenotyping remains a major bottleneck for research activities. In their work, the authors reviewed the literature concerning the disease evaluation methods available for the discovery of the *loci* involved in resistance to downy (*Rpv*) and powdery (*Ren/Run*) mildew. The great variability recorded for environment (from the field to laboratory, from *in vivo* to *in vitro* assays), organs (from whole plant to leaves), rating systems (from discrete to continuous values), and inocula (from field populations to individual isolates) used in different assays highlights the need for a standardization of the methods used for phenotyping. Likewise, the development of affordable and high throughput phenotyping systems is one of the aims for grapevine genetic improvement. Herzog et al. (2022) applied fast sensor technologies for investigating multiple traits (berry impedance, berry texture, and 3D bunch architecture) involved in the interaction between *Botrytis cinerea* (the grey mould agent) and grapevine berries. Impedance of berries, an indirect method for the assessment of cuticle thickness and permeability, was identified as a reliable indicator for disease infection that could be used as a proxy to identify grape varieties resilient to *B. cinerea*.

## Improving grape and wine quality

North American wild grape species show desirable features to counteract biotic and abiotic stresses, but they produce uneven yields and certain off-flavors and aromas that are generally perceived as negative notes by consumers (Vezzulli et al., 2022). Chang et al. (2022) conducted a transcriptomics comparison of two muscadinia (*Muscadinia rotundifolia*) accessions that released candidate genes to account for ripening control and determination of breeding desirable and unfavorable fruit traits present in this species. Besides, Awale et al. (2022) proved the efficiency of a metabolomics-driven approach to explore the genetic basis of wine quality traits in inter-specific hybrids. They identified a series of volatile compounds and volatile precursors characterizing the aroma profile of ‘Cabernet Sauvignon’ and ‘Norton’ grapes and wines, which enabled the full characterization of a breeding hybrid population (*V. aestivalis*-derived cv. ‘Norton’ × *V. vinifera* cv. ‘Cabernet Sauvignon’).

## New resources for the grapevine scientific community

While there is an increasing generation of knowledge on grapevine genomic resources and gene functions that can aid the design of efficient breeding strategies (Delrot et al., 2020), efforts to integrate this information systematically are required for an optimum exploitation of these resources. In a perspective article, Navarro-Payá et al. (2022) present a grape gene reference catalog that is in development by the grapevine research community. Genes Card, a visualization tool gathering gene functions collected in the catalog linked to expression data derived from public transcriptomic datasets, is presented as well and potential breeding-oriented applications of the catalog are discussed.

## Author contributions

GL, PC-B, SLT, and JT co-edited the Research Topic and wrote, edited, and approved this Editorial.

## Funding

JT was funded by a Juan de la Cierva-Incorporación grant (IJC2018-035036-I).

## Acknowledgments

We thank the Frontiers Editorial Office, authors and reviewers for their work in this Research Topic.

## Conflict of interest

The authors declare that the research was conducted in the absence of any commercial or financial relationships that could be construed as a potential conflict of interest.

## Publisher’s note

All claims expressed in this article are solely those of the authors and do not necessarily represent those of their affiliated organizations, or those of the publisher, the editors and the reviewers. Any product that may be evaluated in this article, or claim that may be made by its manufacturer, is not guaranteed or endorsed by the publisher.
